# Supplementary material for: Cutaneous Melanoma: An Overview of Physiological and Therapeutic Aspects and Biotechnological Use of Serine Protease Inhibitors
Source: Molecules. 2024 Aug 16;29(16):3891. doi: 10.3390/molecules29163891 (PMC11357276; doi:10.3390/molecules29163891)
Supplement: Supplementary file 1 [file molecules-29-03891-s001.zip › molecules-3116056-supplementary.pdf]

**Table S1.** Drugs approved by the Food and Drug Administration (FDA) for treating melanoma.

| Type | Drug | Mechanism of Action | Approved for Use |
|---|---|---|---|
| **Chemotherapy** | Aldesleukin | Interleukin (IL)-2, immunostimulant | Approved for melanoma treatment |
| | Binimetinib | BRAF inhibitor | Approved for melanoma treatment |
| | Braftovi (Encorafenib) | BRAF inhibitor | Approved for melanoma treatment |
| | Cobimetinib Fumarate | MEK inhibitor | Approved for melanoma treatment |
| | Cotellic (Cobimetinib Fumarate) | MEK inhibitor | Approved for melanoma treatment |
| | Dabrafenib Mesylate | BRAF inhibitor | Approved for melanoma treatment |
| | Dacarbazine | Alkylating agent, disrupts DNA replication | Approved for melanoma treatment |
| | Encorafenib | BRAF inhibitor | Approved for melanoma treatment |
| | Hepzate (Melphalan Hydrochloride) | Alkylating agent, disrupts DNA replication | Approved for melanoma treatment |

| | | | |
|---|---|---|---|
| | IL-2 (Aldesleukin) | Interleukin (IL)-2, immunostimulant | Approved for melanoma treatment |
| | Imlygic (Talimogene Laherparepvec) | Oncolytic virus, selectively kills cancer cells | Approved for melanoma treatment |
| | Intron A (Recombinant Interferon $\alpha$-2b) | Interferon, immunomodulator | Approved for melanoma treatment |
| | Ipilimumab | CTLA-4 inhibitor, enhances T cell activation | Approved for melanoma treatment |
| | Keytruda (Pembrolizumab) | PD-1 inhibitor, enhances T cell activation | Approved for melanoma treatment |
| | Kimmtrak (Tebentafusp-tebn) | CD3 T-cell receptor binding immunotherapy | Approved for melanoma treatment |
| | Mekinist (Trametinib Dimethylsulfoxide) | MEK inhibitor | Approved for melanoma treatment |
| | Mektovi (Binimetinib) | MEK inhibitor | Approved for melanoma treatment |
| | Melphalan Hydrochloride | Alkylating agent, disrupts DNA replication | Approved for melanoma treatment |
| | Nivolumab | PD-1 inhibitor, enhances T cell activation | Approved for melanoma treatment |

| | | | |
|---|---|---|---|
| | Nivolumab and Relatlimab-rmbw | Combination of PD-1 and LAG-3 inhibitors | Approved for melanoma treatment |
| | Opdivo (Nivolumab) | PD-1 inhibitor, enhances T cell activation | Approved for melanoma treatment |
| | Opdualag (Nivolumab and Relatlimab-rmbw) | Combination of PD-1 and LAG-3 inhibitors | Approved for melanoma treatment |
| | Pembrolizumab | PD-1 inhibitor, enhances T cell activation | Approved for melanoma treatment |
| | Proleukin (Aldesleukin) | Interleukin (IL)-2, immunostimulant | Approved for melanoma treatment |
| | Recombinant Interferon $\alpha$-2b | Interferon, immunomodulator | Approved for melanoma treatment |
| | Tafinlar (Dabrafenib Mesylate) | BRAF inhibitor | Approved for melanoma treatment |
| | Talimogene Laherparepvec (T-VEC) | Oncolytic virus, selectively kills cancer cells | Approved for melanoma treatment |
| | Tebentafusp-tebn | CD3 T-cell receptor binding immunotherapy | Approved for melanoma treatment |
| | Trametinib Dimethyl Sulfoxide | MEK inhibitor | Approved for melanoma treatment |
| | Vemurafenib | BRAF inhibitor | Approved for melanoma treatment |
| | Yervoy (Ipilimumab) | CTLA-4 inhibitor, enhances T cell activation | Approved for melanoma treatment |

| | | | |
|---|---|---|---|
| | Zelboraf (Vemurafenib) | BRAF inhibitor | Approved for melanoma treatment |
| **Topical Treatments** | 5-Fluorouracil (5-FU) | Inhibits thymidine synthesis, leads to defects in genetic material replication, cell death | Topical chemotherapeutic agent |
| | Imiquimod | Immunomodulator, toll-like receptor agonist, releases interleukins, enhances immune response | Adjuvant in melanoma treatment, when surgery is not an option |
| | Ingenol Mebutate | Protein kinase C (PKC) activator, promotes cell death by necrosis, vascular damage | Topical therapy, extracted from Euphorbia peplus |
| | Vitamin A Analogs (Retinoids) | Differentiation of keratinocytes, immunomodulatory effects | Topical therapy |
| **Targeted Anticancer Therapies** | BRAF Inhibitors | Target BRAF mutations, inhibit MAPK pathway | Approved for melanoma treatment, e.g., Vemurafenib, Dabrafenib |
| | MEK Inhibitors | Target MEK mutations, downstream of BRAF, inhibit MAPK pathway | Approved for melanoma treatment, e.g., Binimetinib, Cobimetinib, Trametinib |
| | C-KIT Inhibitors | Target C-KIT mutations | Approved for specific melanoma types, e.g., Imatinib |
| | Multikinase Inhibitors | Target multiple kinases | Approved for specific melanoma types, e.g., Nilotinib, Sunitinib |

| | | | |
|---|---|---|---|
| | CDK4/6 Inhibitors | Target CDK4/6 pathway | Approved for melanoma treatment, e.g., Palbociclib, Abemaciclib, Ribociclib |
| **Immunotherapy** | Interferon-$\alpha$2b | Immunostimulant, interferon | Approved for melanoma treatment |
| | Interleukin IL-2 | Immunostimulant, interleukin | Approved for melanoma treatment |
| | Anti-CTLA-4 (Ipilimumab) | CTLA-4 inhibitor, enhances T cell activation | Approved for melanoma treatment |
| | Anti-PD-1 (Nivolumab, Pembrolizumab) | PD-1 inhibitor, enhances T cell activation, enhances immune-mediated antitumor effects | Approved for melanoma treatment |
| | Anti-LAG-3 (Relatlimab) | LAG-3 inhibitor, enhances T cell activation | Approved for melanoma treatment |
| | Combined Therapy (Ipilimumab and Nivolumab) | Combination of CTLA-4 and PD-1 inhibitors | Approved for melanoma treatment |
| | Combined Therapy (Relatlimab and Nivolumab) | Combination of LAG-3 and PD-1 inhibitors | Approved for melanoma treatment |
| | Oncolytic Virus (T-VEC) | Selectively kills cancer cells | Approved for melanoma treatment |
